# Metabolism of the prodrug lisdexamfetamine dimesylate in human red blood cells from normal and sickle cell disease donors*

**DOI:** 10.3109/21556660.2013.775132

**Published:** 2013-02-13

**Authors:** Michael Pennick

**Affiliations:** Biosciences Department, Shire Pharmaceutical Development Ltd, BasingstokeUK

**Keywords:** Lisdexamfetamine dimesylate, Red blood cell, Sickle cell disease, Enzymatic metabolism

## Abstract

**Objectives:**

Lisdexamfetamine dimesylate (LDX), a long-acting pro-drug psychostimulant, requires conversion to d-amphetamine for therapeutic activity. Conversion of LDX to d-amphetamine occurs primarily in the blood, specifically red blood cells (RBCs). These *in vitro* studies examine potential conversion in blood-containing pathologically deformed RBCs.

**Methods:**

Fresh blood samples from two human male donors with sickle cell disease and two healthy control donors were incubated for up to 4 h with LDX (1 µg/mL) at 37°C. LDX and d-amphetamine were measured by a validated liquid chromatographic mass spectrometric (LC/MS/MS) method.

**Results:**

In incubations of blood from the two donors with sickle cell disease, LDX concentrations declined over time such that 14.1% and 15.3% of initial LDX remained after 4 h. Similarly, in incubations of blood from two healthy donors, LDX concentrations declined over time with 13.1% and 10.5% of initial LDX remaining. Half-life of LDX was 1.30 and 1.36 h for the donors with sickle cell disease and 1.15 and 1.13 h for the healthy donors. Concurrent with the decrease in LDX concentrations, the d-amphetamine concentrations rose in a similar fashion in samples from healthy controls and sickle cell donors. d-Amphetamine levels detected at 4 h with LC/MS/MS were 297.0 ng/mL and 324.3 ng/mL in the two healthy donors and 304.5 ng/mL and 286.6 ng/mL in the two sickle cell donors.

**Conclusions:**

While the current findings are derived from *in vitro* investigations on a small number of samples and the applicability of this *in vitro* experimental system to *in vivo* function has not been established, biotransformation of LDX and the resulting delivery of active d-amphetamine from LDX are likely to be similar in individuals with or without sickle cell disease.

## Introduction

Lisdexamfetamine dimesylate (LDX) is a long-acting pro-drug psychostimulant indicated for the treatment of attention-deficit/hyperactivity disorder (ADHD) in children (6–12 years of age), adolescents (13–17 years of age), and in adults. LDX is a therapeutically inactive molecule. After oral ingestion, it is absorbed as the intact pro-drug and then enzymatically hydrolyzed to l-lysine and active d-amphetamine, which is responsible for the therapeutic effect^1^. The pharmacokinetic characteristics of LDX permit once-daily dosing^2^. Plasma concentrations of d-amphetamine peak at ∼3.0–5.0 h after oral LDX dosing, with a half-life of ∼9.0–10.0 h^2,3^. The process of LDX biotransformation by red blood cells (RBCs) controls the rate of d-amphetamine delivery, unlike other long-acting psychostimulant formulations in which extended activity is dependent on slow tablet dissolution or medication release within the gastrointestinal tract^1^.

The d-amphetamine plasma concentration-time profile is linear and dose proportional and exhibits low inter- and intra-patient variability^3,4^. Moreover, the pharmacokinetic profile of d-amphetamine is relatively similar, regardless of whether LDX is administered orally, intra-nasally, or intravenously^5,6^.

Although it has been erroneously reported that LDX is hydrolyzed in gastrointestinal fluids^7^, the primary site of hydrolytic metabolism of LDX to d-amphetamine is the RBC, but not other human blood fractions^1^. The mechanisms by which RBCs carry out such metabolism are currently being evaluated. RBC-mediated LDX metabolism is robust; the metabolism rate slowly declined with increased dilution, yet was still substantial even when RBC concentrations are reduced to only 10% of normal values^1^. It is not known, however, whether the enzymatic metabolism of LDX occurs on the RBC membrane or within the cell interior. Moreover, the enzyme(s) responsible for the metabolism have not been identified. Peptidases present in RBCs may be candidates for mediating the catalysis of LDX^8^. Given the primary role of RBCs in the hydrolytic metabolism of LDX to d-amphetamine, it is unknown whether pathologic conditions that affect RBCs, such as sickle cell disease, would have an effect on LDX hydrolysis and the resulting pharmacokinetic profile of d-amphetamine. Abnormalities of RBC function that occur in sickle cell disease may affect protein structure and function within an interactive network of RBC proteins^8^.

More detailed information regarding the transport and enzymatic hydrolysis of LDX to d-amphetamine is needed. These *in vitro* studies examined whether enzymatic metabolism of LDX differed in human whole blood samples from healthy donors vs those with sickle cell disease.

## Patients and methods

### Blood samples

#### RBCs

Whole blood samples from two healthy male human donors and from two male human donors with sickle cell disease were purchased from a commercial human blood cell supplier (Bioreclamation, New York, NY).

### *In vitro* enzymatic LDX metabolism assay

To examine the time course of LDX enzymatic metabolism, aliquots (5 mL, in duplicate) of human whole blood were incubated with 50 µL of LDX stock solution (100 µg/mL, leading to a final concentration of 1 µg/mL) at 37°C for 0.0, 0.25, 0.5, 1.0, 2.0, and 4.0 h. Negative control aliquots containing 5 mL 0.1 M phosphate buffer and 50 µL LDX stock solution were incubated in parallel. Incubation reactions were terminated by the addition of chilled acetonitrile (0.5 mL). All samples were extracted by protein precipitation using acetonitrile. Samples were then analyzed and quantified in duplicate for LDX and d-amphetamine using a validated liquid chromatographic mass spectrometric (LC/MS/MS; Triple Quadrupole MS API4000; Applied Biosystems, Sciex; at QPS, LLC, Newark, DE) method.

## Results

### Healthy vs sickle cell disease in human whole blood samples

**Figure 1.**
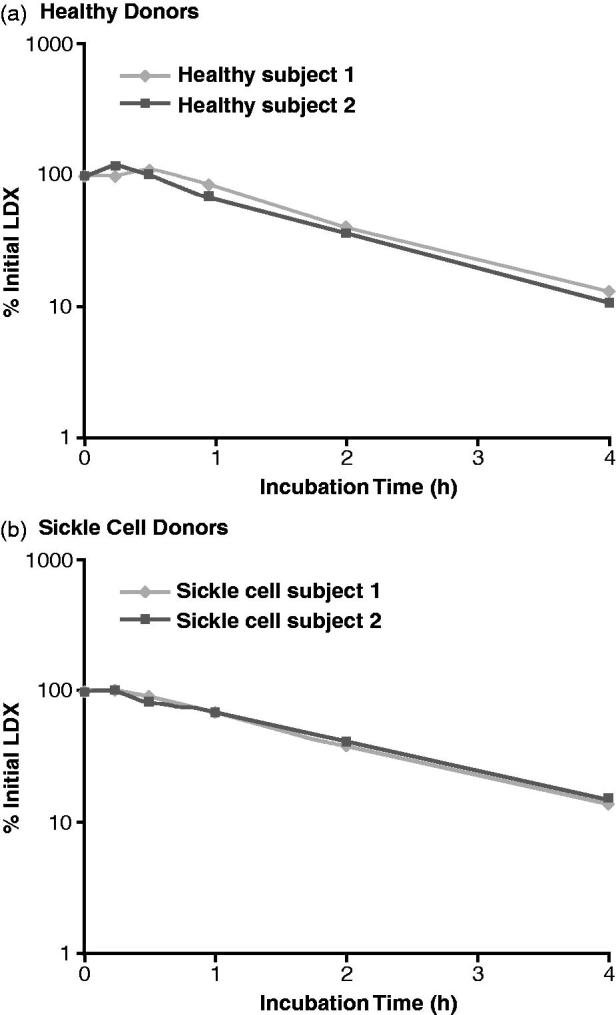
Percentage of initial lisdexamfetamine dimesylate (LDX) concentration during incubation with human whole blood.

The percentages of initial LDX concentration remaining during the 4-h incubation with whole blood from healthy donors and donors with sickle cell disease are illustrated in Figures 1a and b, respectively. At time 0, individual mean LDX concentrations were 823.1 and 668.3 ng/mL for the samples from healthy donors, and 996.0 and 1001.0 ng/mL for the samples from donors with sickle cell disease. After 4 h incubation, individual mean LDX concentrations were 107.6 and 70.0 ng/mL for the samples from healthy donors, and 140.0 and 152.8 ng/mL for the samples from donors with sickle cell disease. With blood from both healthy donors and sickle cell donors, LDX concentrations declined throughout the 4-h incubation period, such that, at 4 h, the LDX concentration had declined to less than 20% of the initial concentration (two healthy donors, 13.1% and 10.5%; two sickle cell donors, 14.1% and 15.3%). The half-life of LDX was 1.15 and 1.13 h for the healthy donors and 1.30 and 1.36 h for the donors with sickle cell disease.

**Figure 2.**
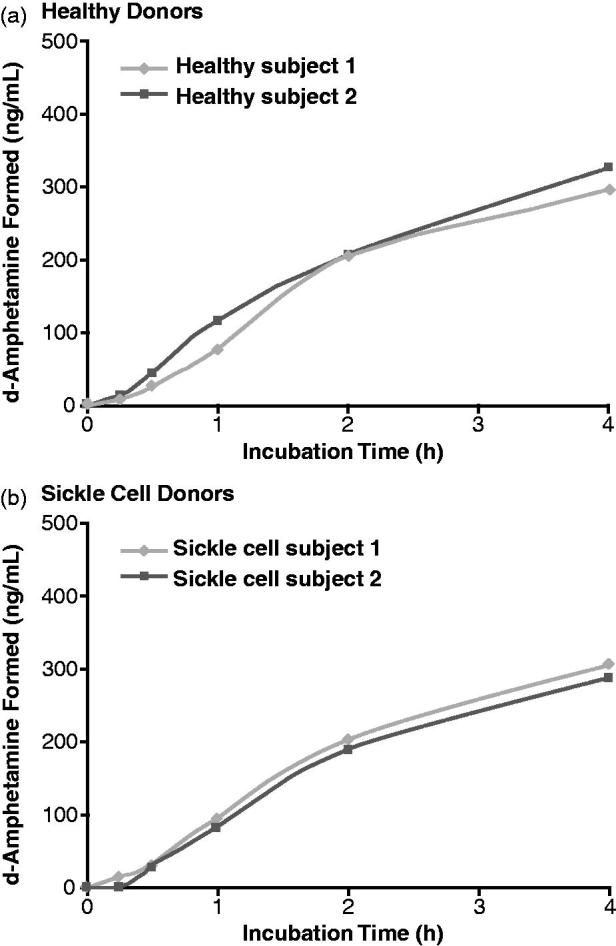
d-Amphetamine concentration during incubation of lisdexamfetamine dimesylate (LDX) with human whole blood.

Figures 2a and b illustrate the concentrations of d-amphetamine observed during the 4-h incubation with whole blood from healthy donors and donors with sickle cell disease, respectively. The d-amphetamine concentrations increased during incubation of LDX in blood samples from both healthy and sickle cell donors. At 4 h, mean d-amphetamine levels detected by LC/MS/MS were 297.0 ng/mL and 324.3 ng/mL in the two healthy donors and 304.5 ng/mL and 286.6 ng/mL in the two sickle cell donors.

## Discussion

In whole blood samples collected from healthy human subjects, hydrolysis of LDX to d-amphetamine was robust. Hydrolysis of LDX was unaffected by RBC sickle cell pathology; the timing and magnitude of LDX metabolism to active d-amphetamine were similar in whole blood samples from donors with or without sickle cell disease.

The current investigation provides support for earlier findings^1^ that the human RBC is an important site of LDX biotransformation to d-amphetamine. In whole blood samples, *in vitro* hydrolysis of LDX to d-amphetamine was robust and unaffected by sickle cell RBC pathology in the current study. Prior *in vitro* work has shown that the rate of LDX hydrolysis is reduced only when RBC concentration is reduced to 10% of normal hematocrit^1^. One might predict from these *in vitro* findings that LDX and d-amphetamine pharmacokinetics in patients with sickle cell disease may be similar to that seen in healthy individuals. In line with this hypothesis, *in vitro* clearance of LDX was similar in blood samples from both healthy and sickle cell donors reported in this study. Nevertheless, there are other sites of pathology in sickle cell disease beyond the RBC (such as the renal system) that may affect LDX clearance. To examine the potential effect of these *in vitro* findings in the clinical setting, it is necessary to directly determine the pharmacokinetics of LDX and d-amphetamine in clinical trials that enroll human participants with sickle cell disease.

It will also be of interest to identify more precisely the mechanism by which RBCs metabolize LDX. Current and previous findings suggest that whichever enzyme(s) are involved, this must be a high-capacity system that is not affected by calcium-dependent enzymes^1^ and may not be significantly altered in settings in which RBC pathology exists. Structurally, LDX may be considered to be a dipeptide and, similar to other such molecules, may interact with transporters, channels, or receptors on RBC membranes^9^.

Prior research has shown that RBCs actively transport catecholamine compounds, such as dopamine, into the cell^10^, which d-amphetamine resembles structurally, putatively via the choline exchange system. Alternately, since RBC surface glycoproteins enzymatically process peptides^9^, LDX may interact with these glycoproteins. Investigation of major RBC enzyme pathways and transporter proteins may be warranted to identify the primary mechanism of LDX biotransformation at this site.

### Limitations

The current findings are derived from *in vitro* investigations on a small number of samples and conducted using commercially obtained blood specimens. The applicability of data from this *in vitro* experimental system to *in vivo* function has not been established. In this study, 4-h LDX blood concentrations in samples from normal donors and those with sickle cell disease were higher than peak plasma concentrations seen in pharmacokinetic studies of healthy volunteers administered doses within the therapeutic range^4^. Biotransformation at lower concentrations has not been studied.

## Conclusions

The *in vitro* biotransformation of LDX to l-lysine and d-amphetamine is similar in human blood from normal donors and those with sickle cell disease. These findings confirm previous results that RBCs are an important site of LDX metabolism in humans. Although the results suggest that the process may be unaffected by sickle cell pathology, it should be noted that *in vitro* studies are not always predictive of the *in vivo* situation, and these findings should be confirmed in clinical studies that include individuals with sickle cell disease. While short- and long-term efficacy and safety of LDX has been characterized in studies in children and adults with ADHD^11–14^, further investigation is needed to identify the precise erythrocyte mechanism(s) involved.

## Transparency

### Declaration of funding

Pre-clinical research was funded by the sponsor, Shire Development LLC, Wayne, PA, USA.

### Declaration of financial/other relationships

MP is a Shire employee and holds stock and/or stock options in Shire.

## Acknowledgments

Under the direction of the author, Michael Pucci, PhD, an employee of SCI Scientific Communications & Information (SCI), Parsippany, NJ and Karen Dougherty, PhD, a former employee of SCI, provided writing assistance for this manuscript. Editorial assistance in formatting, proofreading, copy editing, and fact checking was also provided by SCI. Shire Development LLC provided funding to SCI for support in writing and editing this manuscript. Thomas Babcock, DO, and Brian Scheckner, PharmD, from Shire Development LLC also reviewed and edited the manuscript for scientific accuracy. Assays were performed by QPS, LLC, Newark, DE.
